# Determinants of short birth interval in Ethiopia: A multilevel analysis based on EDHS 2019, Ethiopia, 2023

**DOI:** 10.1371/journal.pone.0311700

**Published:** 2024-10-09

**Authors:** Mulu Tiruneh, Aragaw Tesfaw, Melkalem Mamuye, Desalegn Tesfa, Getaneh Atikilt, Asaye Alamneh Gebeyehu, Wondwosen Teshager

**Affiliations:** Department of Public Health, College of Health Sciences, Debre Tabor University, Debre Tabor, Ethiopia; University of Oulu: Oulun Yliopisto, FINLAND

## Abstract

**Introduction:**

According to the World Health Organization and Ethiopian Demographic and Health Survey on birth spacing, there should be at least a two-year gap between conception and the first of two children born in quick succession. In poor nations like Ethiopia, resource issues were complex, making it difficult to get statistics for the entire country. However, by examining Ethiopian mini demographic and health survey data, we were able to provide data at the national level.

**Method:**

The cross-sectional survey-based study was conducted in several of Ethiopia’s administrative cities and nine regions. In the analysis, sampling weight was used to correct the survey’s non-proportional sample distribution to strata and areas throughout the survey process and restore representative data. The study’s household population was presented and described using descriptive statistics such as weighted frequencies and percentages. The statistically significant factors linked to frequent short birth intervals were found using a multivariable, multilevel logistic regression analysis.

**Result:**

Overall, 4306 weighted multigravida mothers nested within 305 enumeration areas were included in the analysis. The respondents’ mean (standard deviation) of the birth interval was 42.027(26.69). Higher-educated women had 12% lower odds of having a shorter pregnancy (AOR = 0.88; 95% CI: 0.35, 0.98) than women without higher education. The odds of a short birth interval were 3.04 times greater among women in the age category of 40–49 years at first marriage (AOR = 3.04; 95% CI: 1.08, 8.46) than among women in the age category of 15–19 years. This indicates that older women were most likely to have short birth intervals.

**Conclusion:**

In the multilevel logistic regression model, maternal age, maternal educational status, the wealth quintile index, use of contraceptives, duration of breastfeeding, and contextual regions were significantly associated with short birth intervals in Ethiopia.

## Introduction

In reports by the World Health Organization (WHO) and the Ethiopian Demographic and Health Survey (EDHS) on birth spacing, it was advised to wait at least two years between the conception of a child and the birth of that child [1, 2]. Birth spacing is still a problem for reproductive health, as De Jonge *et al*. (2014) claim. Short gestational intervals and greater rates of stillbirth in disadvantaged mothers tend to increase the prenatal death rate [3]. According to a 2017 report, approximately 810 women die each day from pregnancy-and childbirth-related preventable causes. Pregnant women are at a higher risk of complications and death than adolescent women (ages 10 to 14). Ninety-four percent of all maternal deaths occur in low- and middle-income countries [4].

Birth intervals that are too short or too long can affect population growth, fertility rates, and mother and child health [5]. Among the demographic determinants, the survival status of the firstborn children has the greatest influence on the date of the second and third births. As anticipated, the probability of a woman having a second or third child increased dramatically by 2.9 and 2.4 times, respectively, compared to women who had all of their previous children alive [5]. Mihretie *et al*. (2021) assert that optimal birth spacing reduces morbidity and fertility. By providing health information on the advantages of breastfeeding, checking antenatal care during pregnancy, using contraceptives after delivery, and encouraging mothers to make decisions about their health and use of contraceptives to optimize birth spacing for rural communities, we can reduce maternal and infant mortality [6].

According to Titaley *et al*. (2008), short birth intervals resulted in 54.7% of newborn fatalities occurring during the neonatal period, with 29.9% of those deaths occurring on the infant’s first day and 75.6% occurring on the infant’s first week of life [7].

According to Khan *et al*. (2016), maternal and child health issues continue to be the main issue in Ethiopia and the Horn of Africa. Even if efforts have been made to encourage pregnant women to have suitable birth intervals, such as by improving health extension programs and family planning packages [8]. Ethiopia also strives to achieve the ideal birth interval of 24–36 months between two succeeding infants. However, only roughly 32% of children identified their birth interval between 36 and 60 months, with 60% reporting between those two ranges [9].

On the other hand, according to studies by Titaley *et al*. (2008) and Kembo *et al*. (2009), neonatal mortality accounts for nearly 40% of under-five child mortality globally because of the persistence of maternal and child issues linked to premature birth in children whose mothers had no education, but mortality among children is decreased by 24% and 41%, respectively, in children whose mothers had completed primary or secondary education [7, 10].

Even while Ethiopia works hard to lower maternal and infant mortality through health extension initiatives, the goals still haven’t been reached. Numerous studies have shown that 42% of under-five deaths in Africa, including Ethiopia, are due to neonatal mortality [11].

According to earlier studies, there are regional differences in the prevalence of short birth intervals, which range from 6% to 46%, and long birth intervals, which range from 6.1% to 29.5% [12].

The study done in rural Bangladesh indicates that stunting, being underweight, and wasting all had a strong association with short birth intervals [13]. Using contemporary contraceptives, breastfeeding for less than 24 months before the child’s birth, having a female child, and having a low-income index were all strongly related to shorter birth intervals, according to a study done in Arba Minch, South Ethiopia [14, 15].

Additionally, illiterate women, women whose husbands were farmers, and mothers who breastfed their infants for less than a year are all substantially linked to short birth intervals [15, 16]. Additionally, statistically significant associations between inadequate birth spacing and variables like women’s educational status, age at first marriage, distance to the nearest health facility, wealth index, and use of postnatal care following the previous birth were found [15–17].

To ensure that pregnant women have suitable childbearing intervals, efforts have been made to expand family planning services and health extension initiatives. However, the main issue continues to be the short birth intervals of less than two years [8].

In undeveloped countries like Ethiopia, resource issues were complex, making it difficult to collect data at the national level. However, by analyzing Ethiopian Mini Demographic and Health Survey (EMDHS) data, we were able to provide data at the national level. Additionally, it is a severe problem to find out-of-date information that contributes to the failure of future health strategies. Therefore, our study aimed to examine the causes of short birth intervals by applying multilevel analysis techniques.

## Materials and methods

### Study design and data source

The cross-sectional survey-based study was carried out in several of Ethiopia’s administrative cities and nine regions. More than 80 different ethnic groups can be found in Ethiopia, which also has nine regions and two metropolitan areas. For this study, the eleven administrative regions of Ethiopia were divided into three contextual regions: pastoralist (Afar, Benishangul-Gumuz, Somali, Gambella), agrarian (Tigray, Amhara, Oromia, Southern Nations, Nationalities, and Peoples (SNNP)), and urban (Addis Ababa, Dire Dawa, Harari). These contextual regions were defined based on the cultural and socioeconomic backgrounds of their populations [18].

With an estimated population of more than 120 million, Ethiopia is one of the most well-known nations in eastern Africa [19]. Under the general supervision of the Technical Working Group (TWG) and in collaboration with the Federal Ministry of Health (FMOH), the Central Statistical Agency (CSA), and the Ethiopian Public Health Institute (EPHI), one of the data sources used to describe the state of the country’s health is the Ethiopian Demographic Health Survey (EDHS), which is conducted every five years.

### Study population and sampling procedure

We used the KR data set for this study. Women between the ages of 15 and 49 who were either inhabitants of the chosen households permanently or guests who were there the night before the survey were eligible to be questioned. There were 4306 multigravida in the study who gave birth five years before the survey was conducted.

This survey’s sample was designed to provide estimates of important indicators for both the country as a whole and for urban and rural areas individually. In two steps, the sample’s stratification and selection were completed, and urban and rural areas were separated within each region. In each stratum, samples of enumeration areas (EAs) were chosen separately in two steps. At each of the lower administrative levels, implicit stratification and proportional allocation were achieved by classifying the sampling frame within each sampling stratum before sample selection by the administrative units at various levels and by employing a probability proportional to size selection at the first stage of sampling. A total of 305 EAs were chosen proportionally to EA size in the first stage (93 in urban regions and 212 in rural areas). In the second stage, on average, 30 households were randomly chosen from the indicated household sampling frame for each EA. Any additional information regarding the methodology or sampling procedure has been obtained from the 2019 Ethiopian Mini Demographic and Health Survey (EMDHS) report [20].

### Study variables

The length of the birth interval serves as the dependent variable (less than twenty-four months indicates a short birth interval, while higher than or equal to twenty-four months indicates a normal birth interval). Two categories of individual-level and community-level factors each comprised one of the independent variables for this study. The individual-level factors included the mother’s age at the time of the survey, the respondent’s sex, the respondent’s educational level, religion, the wealth quintile index, the child’s sex, the order of birth, the duration of breastfeeding practices, and the use of contraception. When we say the respondent’s sex we mean to say the sex of the household head rather than the sex of participants. So, we tried to show the distribution of sex of household heads as it appears in the EMDHS datasets. In this study, two variables, such as region and place of residence, were taken into account as community-level factors.

### Data management and statistical analysis

Using Stata version 17 software, the data were extracted, recoded, and subjected to additional analyses. Sampling weight is used in analysis to correct non-proportional sample allocation to strata and regions throughout the survey process and to restore representative data. To describe and portray the study household population, descriptive statistics such as weighted frequencies and percentages were used. An investigation of bivariable multilevel logistic regression was first performed. In the multivariable, multilevel logistic regression analysis, factors having a p-value of less than 0.25 were included. The determination of statistically relevant variables linked to short birth intervals was done using a multivariable, multilevel logistic regression analysis.

We fitted four alternative models after choosing the pertinent factors for the outcome variable. The second model included the impact of the individual-level variables and examined the duration of the birth interval on individual-level factors that affect it, in contrast to the null model, which did not include any explanatory variables and just included a random intercept. The final model examined the effects of individual-level and community-level variables, which examine the duration of the birth interval with both individual factors and community-level factors. The third model included the influence of community-level variables, which examined the birth interval’s duration with cluster-based factors influencing it.

Using the intraclass correlation (ICC), median odds ratio (MOR), and proportional change in variation (PCV), the variation in the short birth interval was assessed. A group’s or cluster’s level of similarity is gauged by the ICC. If the ICC value is greater than 10, multilevel logistic regression analysis is used instead of standard logistic analysis to account for the data’s hierarchical nature. The intraclass correlation was calculated using the following formula;

$$ICC=\frac{{\delta^{2}}_{U0}}{{\delta^{2}}_{U0}+{\delta^{2}}_{\varepsilon }}$$

were:

$${\delta^{2}}_{\varepsilon }=\frac{\pi^{2}}{3}$$

is individual level, and *δ*^2^_*U*0_ cluster variances

A MOR is the median of a set of odds ratios between an individual chosen at random from two clusters and those in a higher-risk and lower-risk cluster. It gauges the degree of birth interval length difference between clusters. If the MOR value is greater than 1, it indicates that there is variation between clusters. We computed the MOR value using the following equation;

$$MOR=exp\left(\sqrt{2*V_{A}}*0.6745\right)\approx exp\left(0.95\sqrt{V_{A}}\right)$$

Where: V_A is the cluster level variance, and 0.6475 is the 75th commutative distribution [21]. Proportional change in variance (PCV) measures the explained variance difference in the final model by combining individual and community-level factors [22].

Finally, we can calculate the PCV value using the following formula;

$$PCV=\frac{var\;(reduced\;model-full\;model)}{var(reduced\;model)}*100$$

Given that all three metrics measure model fitness, the best model among the four fitted models was selected using the Akaike information criteria (smallest AIC), likelihood ratio (highest LL), and deviance (smallest). The strength of the association between independent variables and the length of the birth interval was assessed using an adjusted odds ratio with a 95% confidence interval. A p-value of 0.05 or less was regarded as statistically significant.

### Ethical consideration

Datasets for the EMDHS 2019 have been downloaded from the Demographic and Health Survey website after receiving the required download permissions. The DHS public-use dataset’s IRB-approved procedures make it impossible for respondents, households, or sample communities to be identified in any way. The data files do not contain names of people or addresses of households. By keeping the data from a third party and using the retrieved data just for the study’s purposes, the confidentiality of the data was maintained.

## Result

### Sociodemographic characteristics of the study participants

The study included 4306 weighted multigravida who were distributed among 305 enumeration areas. The respondents’ average age (standard deviation) was determined to be 30.67 (6.03). Only 2.07% of the study’s total participants had a background in higher education. Among the total respondents, 63% (2711) did not use any kind of contraception, while nearly two-thirds (37%) of the respondents were used contraceptive. 44.8% of all participants whose data were examined fell into the 30- to 39-year-old age group. The median time between births in this study was 35.8 months, and the mean (standard deviation) of the birth interval was 42.027(26.69), while the prevalence of the short birth interval was 25.67% (1105). Based on EMDHS data, the majority of study participants (85.56%) reside in the agrarian region, with 77.47% of total participants living in rural Ethiopia. Of the total participants, 2209(51.3%) were not currently breastfeeding their child. More than forty-two percent of the respondents were still breastfeeding their child. Based on our finding 1146(26.6%) respondents were poorest and 656(15.2%) were richest while 809(18.8%) respondents have middle income (Table 1).

**Table 1:** Frequency of individual-level and community-level factors in EMDHS (2019), Ethiopia (n = 4306).

Variables	Categories	Weighted frequency	Weighted (%)
Sex	Male	3759	87.30
	Female	547	12.70
Educational status	No education	2683	62.32
	Primary	1304	30.28
	Secondary	230	5.34
	Higher	89	2.07
Age category	15–19	41	0.95
	20–29	1863	43.26
	30–39	1929	44.80
	40–49	473	10.99
Sex of the preceding child	Male	2203	51.16
	Female	2103	48.84
Religion	Orthodox	1368	31.77
	Muslim	1731	40.20
	Protestant	1115	25.9
	Others	92	2.13
Wealth status	Poorest	1146	26.61
	Poorer	979	22.74
	Middle	809	18.79
	Richer	716	16.63
	Richest	656	15.23
Order of Birth	2–3	1748	40.60
	4–6	1725	40.06
	7+	833	19.34
Use of contraceptive	Modern	1,566	36.36
	Traditional	29	0.67
	Not used	2,711	62.97
duration of breastfeeding	Not currently breastfeeding	2,209	51.30
	Never breast feeding	283	6.56
	Still breastfeeding	1,814	42.14
Place of residence	Urban	970	22.53
	Rural	3336	77.47
Region	Pastoralist	498	11.56
	Agrarian	3684	85.56
	Urban setting	124	2.88

### Factors of the short birth interval among reproductive women

Maternal age, maternal educational status, wealth quintile, usage of contraceptives, length of breastfeeding, and contextual regions were associated with short birth intervals in the final model, which includes individual-level and community-level characteristics.

When compared to women in the age group of 15 to 19 years, the odds of having a short birth interval were 2.75 times greater for women in the 30 to 39-year age group at the time of their first marriage (AOR = 2.75; 95% CI: 1.03, 7.32). Similarly, when the other factor variable was held constant, the odds of a short birth interval were 3.04 times greater among women in the age category of 40–49 years at first marriage (AOR = 3.04; 95% CI: 1.08, 8.46) than among women in the age category of 15–19 years. Higher-educated women had 12% lower odds of having a shorter pregnancy (AOR = 0.88; 95% CI: 0.35, 0.98) than women without higher education. Hence, women who have higher education levels have better or normal birth intervals but, a woman who has a low education level is exposed to short birth intervals. Compared to the poorest families, women from the poorer households had odds of having a short birth interval that was 41% higher (AOR = 1.41; 95% CI: 1.03, 1.91) while keeping all individual and community factor variables constant. The odds of a short birth interval were also 70% greater in affluent households (AOR = 1.70; 95% CI: 1.04, 2.77) than in poorer households. Similarly, when controlling for other component variables, the odds of a short birth interval were 56% greater in the richest households (AOR = 1.56; 95% CI: 1.11, 2.70) than in the poorest households. This could be because women in the better wealth quintile index have access to healthcare information, knowledge, and reasonably priced healthcare services, as well as affordable resources for education and other social well-being.

Women who utilized traditional means of contraception were 68% (AOR = 0.32; 95% CI: 0.13, 0.80) less likely to use contraceptives than women who did not use any contraceptive methods after controlling for all other contributing factors. This indicates that women who take contraceptive mechanisms had a normal birth interval than women who did not use any kind of contraception. Women who breastfed up until the time of the survey were 62% (AOR = 0.38; 95% CI: 0.28, 0.82) less likely to experience a shorter postpartum period than women who do not currently breastfeed.

Concerning the region, women residing in the agrarian region were 59% (AOR = 0.41; 95% CI: 0.2, 0.95) less likely to have subsequent births than women residing in pastoralist regions by holding other factors constant.

In summary, the respondent’s age and wealth status of the respondents were more likely to have short birth intervals while, the educational status (being higher education level) of the respondents, usage of contraceptives, duration of breastfeeding, and women who live in the agrarian region were less likely to have a short birth interval (Table 2).

**Table 2:** Multilevel analysis for individual-level factors and community-level factors of the short birth interval using EMDHS 2019, Ethiopia (n = 4306).

Variables	Model II	Model III	Model IV
	AOR (95% CI)	AOR (95% CI)	AOR (95% CI)
Sex (**Reference Female**)			
Male	1.42(1.02, 2.00)		1.37(0.97, 1.94)
Educational status (**Reference no education**)			
Primary	1.23(0.93, 1.62)		1.21 (0.91, 1.60)
Secondary	1.10(0.58, 2.11)		1.11(0.57, 2.14)
Higher	0.85(0.35, 0.92)		**0.88(0.45, 0.98)**
Age category **(Reference 15–19)**			
20–29	1.73(0.66, 4.57)		1.71(0.65, 4.53)
30–39	2.82(1.07, 7.47)		**2.75 (1.03, 7.32)**
40–49	3.14(1.13, 8.69)		**3.04(1.08, 8.46)**
Religion **(Reference Orthodox)**			
Muslim	0.52(0.38, 0.72)		0.58 (0.56, 1.38)
Protestant	0.69(0.46, 1.03)		0.69(0.47, 1.04)
Others	0.63(0.33, 1.21)		0.62 (0.32, 1.21)
Wealth status (**Reference poorest**)			
Poorer	1.47(1.09, 1.99)		**1.41(1.03, 1.91)**
Middle	1.37(0.95, 1.99)		1.31(0.91, 1.91)
Richer	1.79(1.12, 2.86)		**1.70 (1.04, 2.77)**
Richest	1.66(1.11, 2.5)		**1.56(1.11, 2.70)**
Use of contraceptive **(Reference not used)**			
Traditional	0.32(0.13, 0.79)		**0.32(0.13, 0.80)**
Modern	1.05(0.84, 1.31)		1.03(0.82, 1.29)
Duration of breastfeeding **(Reference Not currently breastfeeding)**			
Never breast feeding	0.71(0.47, 1.06)		0.70(0.47, 1.06)
Still breastfeeding	0.8 (0.14, 0.97)		**0.38 (0.28, 0.82)**
Place of residence **(Reference Rural)**			
Urban		1.33(0.94, 1.90)	1.08(0.69, 1.7)
Region **(Reference pastoralist)**			
Agrarian		0.69(0.10, 0.86)	**0.41(0.2, 0.95)**
Urban setting		0.78(0.21, 0.92)	1.17(0.71, 1.93)

**AOR** = Adjusted odds ratio; **CI** = confidence interval; **Model II** = adjusted for individual level factor; **Model III** = adjusted for community level factors; **Model IV** = final model adjusted for individual and community level factors; **Others** = catholic/traditional/other

### Multilevel analysis

The birth interval probabilities differed between communities in a statistically significant way (p < 0.001) in the null model findings. The null model suggested that the variation between communities accounted for 14.9% of the overall variance in the distribution of short birth intervals (ICC = 0.149). The proportionate change in variance demonstrated that a higher proportion of the variation in the short birth interval was explained by the addition of factors to the null model. Similar to this, the entire model’s ICC values showed a higher PCV value, indicating that the combined components could account for 51.7% of the variation in short birth intervals (at individual and community levels). The MOR in the null model was 2.05, which indicated that there were differences between communities in the recent birth interval because the MOR was higher than one. After all covariates were included in the null model, the unaccounted-for community difference in short birth intervals decreased to a MOR of 1.65. This shows that even after taking individual and community-level factors into account, the effect of clustering is statistically significant in the final model.

Only individual factors that have a p-value of less than or equal to 0.25 and affect short birth intervals were added to Model II. As a result, the respondent’s sex, maternal age, educational level, religion, wealth quintile, use of contraceptives, and the length of breastfeeding period were all considered potential model factors.

Only community-level variables with an addition p-value less than or equal to 0.25 were considered candidate variables in Model III.

The final model, Model IV, includes both community-level and individual-level factors. Model IV was the model that fitted the data best, based on the values of Akaike’s Information Criterion (AIC) (lowest), Deviance (smallest), and loglikelihood (highest). Model IV was the last model included in the analysis and utilized to interpret the results of this investigation.

In summary, we fitted four different models namely, the null model (which did not include any explanatory variables and just included a random intercept), the second model (examined individual-level variables), the third model (examined community-level variables), and the fourth model (examined both individual and community level variables) after choosing the pertinent factors for the outcome variable. We assessed the variation in the short birth interval by using intraclass correlation (ICC), median odds ratio (MOR), and proportional change in variation (PCV). In addition to this, we compared the four models by using AIC, Deviance, and Log-likelihood, and we got model IV was the last model included in the analysis and utilized to interpret the results of this investigation (Table 3).

**Table 3:** Results from random intercept model (a measure of variation) for the short birth interval at cluster level using multilevel logistic regression analysis using EMDHS 2019, Ethiopia, 2023(n = 4306).

Measure of variations	Null model	Model II	Model III	Model IV
MOR	2.05	1.67	1.88	1.65
ICC (%)	14.95	8	11.8	8
PCV (%)	Reference	50	24.14	51.7
Model fit				
AIC	4689.702	4586.667	4657.713	4538.826
Deviance	4685.702	4546.668	4647.7132	4542.8256
Loglikelihood	-2342.851	-2273.334	-2323.8566	-2271.4128

## Discussion

This study aimed to find birth interval predictors across Ethiopian regions using data from the 2019 Ethiopian Mini Demographic and Health Survey. According to the findings of this survey, around 25.67% of Ethiopian women had short birth intervals. This finding is consistent with research by Zegeye *et al*. (2020) [23], approximately similar to the study conducted in Northern Ethiopia (23.3%) [24], is somewhat comparable to a study done in Jima, Southwest Ethiopia (27%) [25], and is different from a previous study by Desalegn et al. (2020) that used the EDHS, 2016 and found that the prevalence of this report is around 45.7% [26]. Additionally, the current study differs from the earlier one that was carried out in a developing region of Ethiopia (46%) [27]. The difference between the definitions or classifications for the short birth interval may be the cause of this disagreement. The WHO guideline [2] took into account a birth interval of 33 months, whereas our study took into account a birth interval of less than 24 months and a short birth interval based on the EDHS recommendation. According to the present study, the median time between births for children who weren’t born within the five years before the survey was 35.8 months. This result is roughly comparable to earlier research conducted in Rufiji, Tanzania, where the median birth interval was 33.4 months [28].

According to our study, individual- and community-level characteristics were substantially correlated with short birth spacing. Short birth intervals were substantially correlated with maternal age (AOR = 2.75; 95% CI: 1.03, 7.32), educational level (AOR = 0.88; 95% CI: 0.35, 0.98), wealth quintile (AOR = 1.70; 95% CI: 1.04, 2.77), usage of contraception (AOR = 0.32; 95% CI: 0.13, 0.80), and length of feeding at the individual level (AOR = 0.38; 95% CI: 0.28, 0.82). Short birth intervals were associated with contextual regions at the community level (AOR = 0.41; 95% CI: 0.2, 0.95).

According to the study’s findings, women with a higher level of education had a 12% lower likelihood of having short births than women with no formal education. This means a mother who has a higher education level has less risk of short birth interval or a mother who has a low education level is exposed to short birth interval. This finding is supported by studies conducted in Tanzania [28]; the Democratic Republic of the Congo [29], Southern Ethiopia [30]; and Arba Minch District of Ethiopia [14]. This may occur as a result of mothers becoming more aware of the effects of many births on both maternal and child health as their level of education improves. Therefore, compared to women with no education, those with higher levels of education had a lower probability of having short birth intervals.

According to the current study, women in the age groups of 30 to 39 and 40 to 49 in their first marriage had higher probabilities of having a short birth interval than women in the 15- to 19-year-old age group. This could be due to as age increases the ability of women to produce their offspring would be decreased. Therefore, Women who did not have children at a young age are worried that they will not have children as they get older, so their desire to have children increases as they get older. This finding is supported by the study done in Ethiopia [26]. Birth spacing is affected by marriage age, according to a prior study by MacQuarrie et al. (2016) [31]. On the other hand, in the earlier Ethiopian research, age categories older than 30 years did not correlate with frequent births [23]. This mismatch can be brought on by the distinct research design and the use of various age classifications. The new study limits the data from the Mini Ethiopian Demographic and Health Survey, which may be another difference between it and the prior study.

Short birth intervals were statistically significantly influenced by the household wealth quintile index. Compared to women who had the lowest economic position, women with the poorest economic status had a higher likelihood of having a short childbearing interval. Numerous studies suggested that the wealth index offered reliable birth interval predictions. The current study was consistent with other studies carried out in Saudi Arabia [32], Lemo, District Ethiopia, [33], and Ethiopia utilizing EDHS data [26]. This could be because women in the better wealth quintile index have access to healthcare information, knowledge, and reasonably priced healthcare services, as well as resources and education.

According to our research, women who breastfed up to the survey period had a 62% lower chance of having a baby quickly than those who did not. This finding is supported by research from Serbo, Southwest Ethiopia [34], Northern Ethiopia [24], Dodota district, Southern Ethiopia [35], a study conducted in Ethiopia [23], and Northern Ethiopia [36]. The ideal breastfeeding period lengthens the interval between two subsequent deliveries. This indicates that the chance of a short birth interval for the subsequent birth decreases as the woman’s feeding habit increases. Women who take contraceptives through traditional mechanisms had a 68% lower chance of having a baby quickly than women who did not use any kind of contraception. A study conducted in southern Ethiopia supports this conclusion [37]. Contraceptive usage has significantly contributed to the low percentage of mothers with short gestation periods, even though this method of birth control is the conventional one.

Women in pastoralist regions had a 59% higher likelihood of having a second child than women in agrarian regions, according to the present study. This finding is supported by research conducted in Southwest Ethiopia’s Jimma Zone [26], Eastern Sudan’s Kassala [38], and Southwest Ethiopia [25]. Women’s experiences with short birth intervals are influenced differently by the characteristics of the communities where they live. Therefore, women who live in a distinct contextual region, such as an agrarian region, are less likely to suffer a short birth gap. This could occur because of the relatively active lifestyle and high cost of living in agrarian regions, which prompts women to delay having children and engage in income-generating activities to make ends meet and support their families.

The following were the limitations of this investigation. Initially, the birth interval data may be subject to recall bias due to its retrospective recording. Second, no causal implications can be drawn because the analyses were performed using data from a cross-sectional survey. Therefore, prospective research is required.

## Conclusion

According to the study, around 25% of Ethiopian women give birth frequently. Both community- and individual-level variables may have a role in this short birth interval. In the multivariable, multilevel logistic regression model, maternal age (with age categories 30 to 39 and 40 to 49), maternal educational status, the wealth quintile index, use of contraceptives, duration of breastfeeding, and contextual regions were significantly associated with short birth intervals in Ethiopia. As we have indicated in our findings older women were most likely to have short birth intervals. Similarly, a mother who has a higher education level has less risk of a short birth interval, or, a mother who has a low education level is exposed to a short birth interval. Women who take contraceptives have a lower chance of having a baby quickly than women who do not use any kind of contraception. The ideal breastfeeding period could affect the interval between two subsequent deliveries. This indicates that when women increase their breastfeeding habits the chance of a short birth interval for the subsequent birth becomes optimal. After adjusting for the impacts of both individual- and community-level characteristics, the measure of variation (random-effects) analysis result showed that cluster effects were still significant, indicating that the community context influences how women experience the short birth interval. The study also found that where women live has a significant impact on their decision to postpone having children. This suggests that the area played a key role in determining the birth period before. To increase pregnant women’s ideal birth intervals, the Ethiopian government should expand family planning access and boost health extension initiatives. The Ministry of Health should collaborate with the Ministry of Education to enhance the educational standing of women in the community, particularly in the pastoralist zone because there was a substantial correlation between educational status and women’s experiences of the brief birth gap.

## Abbreviations

AOR: Adjusted odds ratio
EA: Enumeration area
EMDHS: Ethiopian mini demographic and health survey
ICC: Intra class correlation
MOR: median odds ratio
PCV: Proportional change in variance
SNNP: South Nation Nationalities and People
WHO: World Health Organization
